# Quantifying Age-Related Rates of Social Contact Using Diaries in a Rural Coastal Population of Kenya

**DOI:** 10.1371/journal.pone.0104786

**Published:** 2014-08-15

**Authors:** Moses Chapa Kiti, Timothy Muiruri Kinyanjui, Dorothy Chelagat Koech, Patrick Kiio Munywoki, Graham Francis Medley, David James Nokes

**Affiliations:** 1 KEMRI-Wellcome Trust Research Programme, Kilifi, Kenya; 2 Mathematics and WIDER, University of Warwick, Coventry, United Kingdom; 3 School of Life Sciences and WIDER, University of Warwick, Coventry, United Kingdom; Kenya Medical Research Institute - Wellcome Trust Research Programme, Kenya

## Abstract

**Background:**

Improved understanding and quantification of social contact patterns that govern the transmission dynamics of respiratory viral infections has utility in the design of preventative and control measures such as vaccination and social distancing. The objective of this study was to quantify an age-specific matrix of contact rates for a predominantly rural low-income population that would support transmission dynamic modeling of respiratory viruses.

**Methods and Findings:**

From the population register of the Kilifi Health and Demographic Surveillance System, coastal Kenya, 150 individuals per age group (<1, 1–5, 6–15, 16–19, 20–49, 50 and above, in years) were selected by stratified random sampling and requested to complete a day long paper diary of physical contacts (e.g. touch or embrace). The sample was stratified by residence (rural-to-semiurban), month (August 2011 to January 2012, spanning seasonal changes in socio-cultural activities), and day of week. Usable diary responses were obtained from 568 individuals (∼50% of expected). The mean number of contacts per person per day was 17.7 (95% CI 16.7–18.7). Infants reported the lowest contact rates (mean 13.9, 95% CI 12.1–15.7), while primary school students (6–15 years) reported the highest (mean 20.1, 95% CI 18.0–22.2). Rates of contact were higher within groups of similar age (assortative), particularly within the primary school students and adults (20–49 years). Adults and older participants (>50 years) exhibited the highest inter-generational contacts. Rural contact rates were higher than semiurban (18.8 vs 15.6, p = 0.002), with rural primary school students having twice as many assortative contacts as their semiurban peers.

**Conclusions and Significance:**

This is the first age-specific contact matrix to be defined for tropical Sub-Saharan Africa and has utility in age-structured models to assess the potential impact of interventions for directly transmitted respiratory infections.

## Introduction

Interventions for the prevention or control of infectious diseases are better formulated on the basis of a quantitative understanding of the determinants of the spread of infection within a population. In the case of directly transmitted respiratory viruses, such as influenza viruses and respiratory syncytial virus (RSV), transmission is effected through interaction or contact between individuals sufficiently close for virus to pass from one person to the next. It follows that the transmission dynamics of these viruses are determined by the structure and rates of such contacts between susceptible and infectious individuals in a population. Mathematical models of infectious disease transmission are recognized as important tools for exploring the potential impact of interventions [1], [2]. To capture greater reality these models generally incorporate age as the key structural feature governing transmission patterns [3], [4]. Increasingly the models designed for the study of respiratory infections utilize direct estimates of contact rates within and between age groups of a population by which to determine who acquires infection from whom [3], [5]–[8].

The source of direct estimates of contacts is usually the self-completed diary and follows the early work by Edmunds *et al* [9]. A sample of the population under study is selected to complete a record of each of the contacts made by the participant with other individuals on a chosen day. These diaries usually aim to collect data on the age of the participant and the ages of all individuals contacted, stratified by the intensity of the contact encounter (usually conversation and touch) [10], the frequency of contact with the same individual or the total duration of this pair-wise contact in the day, the location or context in which the interaction occurs [11], [12], and the day of the week [13], [14]. There are inherent problems with diary collected data including failure to record all contacts and difficulty in comprehending the process of completion. Measures taken to minimize resultant error and bias include recap interviews on collection of diaries and provision of a ‘shadow’ to record the contact data for very young or illiterate participants [7].

Contact diary data reflect the social, behavioural and demographic characteristics of the study population, which may vary from location to location. Specifically, there will be variation between locations in population density, age structure, household occupancy, work practices, schooling, religious gatherings and transport, all of which may have a bearing on the patterns and rates of contact and hence the spread of respiratory infection. The majority of contact diary-based studies have been conducted in developed countries, and only two have been in low income settings, one in an informal urban settlement in South Africa [7] and the other in a semi-rural community in Vietnam [8]. Given all of the above there is a need to characterize contact patterns more widely, particularly in low income communities where least is known.

We aimed to define and quantify an age-specific matrix of rates of contact between individuals within a rural Kenyan population for the purpose of generating data suitable for the mathematical modelling of the transmission dynamics of respiratory syncytial virus by which to assess the impact of vaccine intervention strategies.

## Methods

### Study area

**Figure 1:**
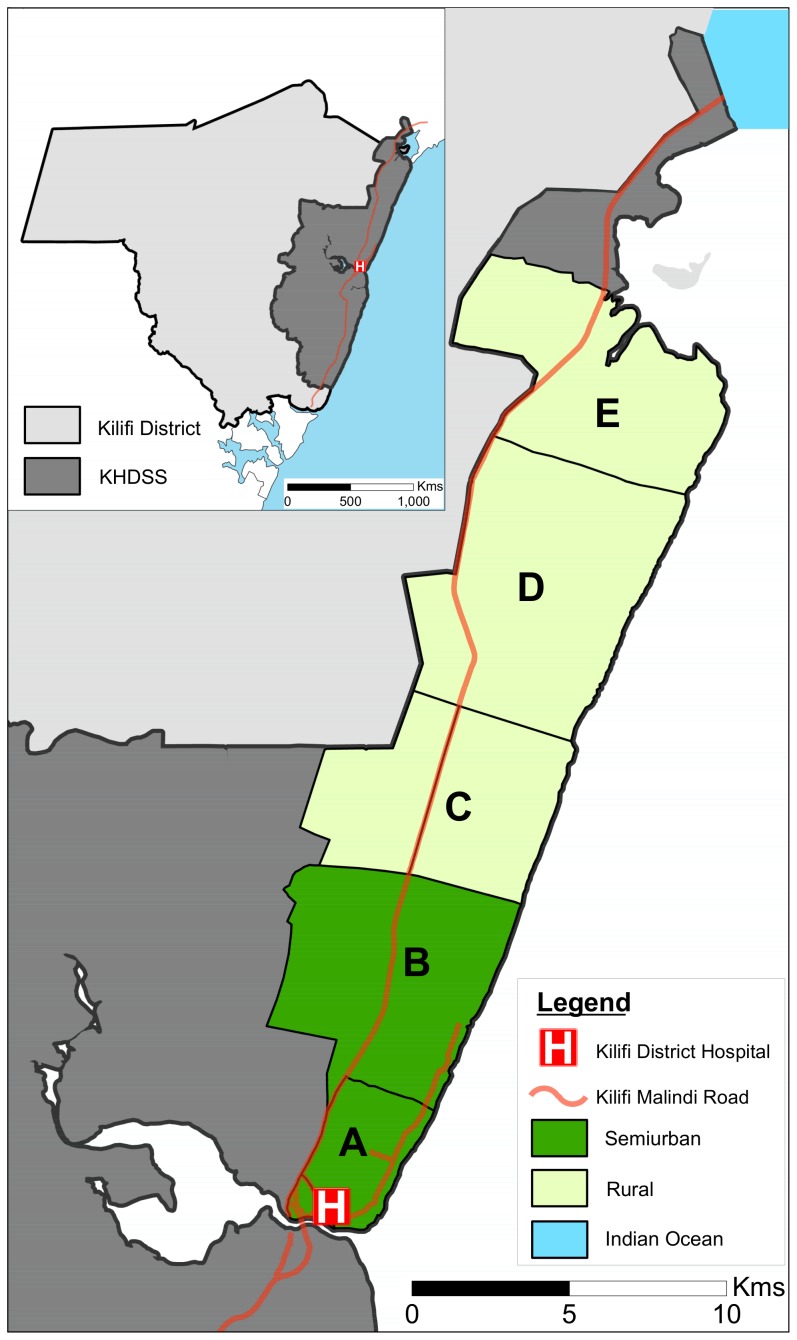
Map of the study area. The inset shows the location of the KHDSS in relation to the former Kilifi District (part of Kilifi County). The study area locations are conventionally categorised as semiurban (Kilifi Township [denoted A] and Tezo [B]), and rural (Ngerenya [C], Roka [D] and Matsangoni [E]).

The study was conducted in 5 locations in the northern part of the Kilifi Health and Demographic Surveillance System (KHDSS). The locations were categorised as semiurban (Kilifi Township [denoted A] and Tezo [B]) and rural (Ngerenya [C], Roka [D] and Matsangoni [E]) as portrayed in Figure 1. The categorisation into semiurban and rural areas is similar to that used by Molyneux *et al* [15]. In March 2011 the KHDSS had a population of 261,919 with mean age of 21.8 and 21.1 years in semiurban and rural areas, respectively. Mean population density in semiurban and rural areas was 530 and 360 people/km^2^, respectively. The average household size was higher in the rural compared to semiurban areas (9.2 versus 7.0, respectively) and about a fifth of the population was below 5 years of age. The KHDSS is described further by Scott *et al* [16].

### Study design

Participants were chosen at random from enumeration registers for each of the five locations (in proportion to location size) and in equal number from 6 age groups assumed to approximate to key social or behavioural groups: <1 (infants), 1–5 (pre-school), 6–15 (primary school), 16–19 (secondary school), 20–49 (adults), and >50 (elderly) years. Recruitment was staggered over a six-month period (Aug 2011 to Jan 2012). All residents who gave informed consent or for whom informed consent was given by their parents, and who were planning to stay in the KHDSS for at least three months were included.

Sample size of the study was based on an estimate of the contact rate variation (SD = 13) from an unpublished contact diary school study (n = 177) recently undertaken in the KHDSS. Using standard methods [17] a required sample size of 150 individuals in each of the six age groups (ie 900 over all age groups) was determined to give an estimate with a 95 percent confidence interval (95% CI). To account for possible non-response and errors in diary completion, this number was scaled up by 20% to give a final sample size of 1,080 individuals.

A contact person was defined as someone with whom the participant had a direct physical encounter (a “contact”), and involved direct skin-to-skin touch such as embracing, kissing or shaking hands. Each contact was recorded only once in the diary during the day of study, and repeat encounters were recorded as tallies. Participants were expected to keep the diary for a day, defined as the period between first waking and going to bed for the night. Participants were assigned a day of the week for completing a contact diary by block randomisation.

### Study implementation

Five focus group discussions were scheduled within the study area to assess the feasibility and suitability of using the diaries. The groups were composed of primary school students (class 4–8, approximate age range 10–17 y), secondary school students (form 1–4, age range 15–21 y), kindergarten teachers (age range 23–55) and separate male and female groups of Kenya Medical Research Institute (KEMRI) Community Representatives (age range 20–50 y) [18]. A pilot study was conducted in the first month among 50 participants to assess the ease of understanding the diary, and to validate an exit interview to be undertaken on collection of the diary from the participant for verification of the entries. From this, we adopted a text and pictorial diary translated from English to Swahili and Giriama (local dialect). The diaries incorporated the age-class of the persons contacted and frequency of the contacts made (Figure S1).

Each eligible participant was approached by a trained fieldworker to gain consent, train in use of the diary, select day of study, for diary collection and exit interview. All participants under 10 years old and other individuals who were unable to read and write (established by asking literacy status of individuals aged over 10 years) selected a “shadow” to record the participant's daily contacts. The shadow was someone who spent most time with the participant and would be in a position to record the contact details of the participant at regular intervals. Shadows were trained on how to keep the diary on behalf of the selected participant, and requested not to influence the normal behaviour of the participant. An alarm wrist watch was lent to each participant or shadow for the duration of study and pre-set to go off at hourly intervals providing a prompt to record recent contacts either directly in the diary or in a paper reminder table prior to transferring the data to the diary at a convenient time. One day prior to the selected day, the fieldworker visited the participant (and shadow) for training and allocation of study material (diary, pen, watch, reminder table). The fieldworkers also recorded the socio-demographic information about the participant (occupation, number of years of completed education, family composition, sleeping arrangements i.e. sharing of bedroom or bed) using a questionnaire (Figure S2).

On the appointed study day, for each different individual physically contacted, participants recorded the assumed age class of the person contacted in the diary against a unique identity (ID) code. The fieldworker revisited the participant at most 48 hours after the diary-keeping to verify the recorded details as actual events, and to fill in a questionnaire (Figure S3) on the participant's experiences, e.g. difficulty encountered, and whether all contacts were recorded or the diary induced a behaviour change such as increasing number of physical contacts. Fieldworkers also recorded whether the contact was known to the participant to assess familiarity of contacts, as well as the frequency of usual contacts with this individual (daily or almost daily, once or twice a week, once or twice a month, or less than once a month). After successful data collection, participants (and shadows) aged 18 years and over were given 3.5 US dollars as compensation for their time, while school going students were given a stationery pack containing items of similar value.

### Data analysis

The primary outcome was age-specific mean number of contacts per person per day, 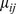 (henceforth referred to as contact rate). Let indices 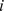 and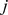 represent age groups, such that 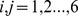, corresponding to <1, 1–5, 6–15, 16–19, 20–49, ≥50 years, respectively. Further, let 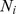 be the total number of participants in age group 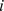 such that 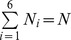, the total number of participants in the study. Let 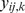 be the number of contacts that participant 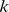 in age group 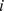 has with respondents in age group 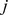. Then, the total number of contacts, denoted 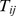 is given by 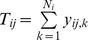. Therefore, the daily contact rate per individual of age group 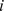 with individuals of age group 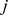 is 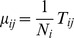.

Differences in the mean contact rates for each covariate (gender, age group, presence of a shadow, season, residence and day of week) were assessed using analysis of variance (ANOVA). The uncertainty of the contact rate estimates was summarised by generating a 95% Confidence Interval (CI) through 2,000 non-parametric bootstraps as described by Carpenter *et al* [19]. Further analysis involved computing weights to eliminate possible selection bias within the semiurban-rural sample compared to KHDSS population (see Text S1).

### Ethical review and consent

The Kenya Ethical Review Committee (KEMRI/RES/7/3/1) and the Biomedical and Social Ethics Review Committee of the University of Warwick (134-07-2011) approved the study. Written informed consent was sought from participants (and shadow) aged ≥18 years and from parents or guardians for those aged <18 years.

## Results

### Baseline characteristics

The study took place over the period 17th August 2011 to 31^st^ January 2012. 1,080 individuals were randomly selected from the KHDSS register, with an additional 58 individuals randomly selected to replace those who refused to give consent. Of the 1,138 individuals no consent was obtained for 515 (45%) for the reasons detailed in Table S1. Of the 623 (55%) who agreed to participate in the study, 606 diaries were collected by the end of the study period, of which 38 were discarded due to discrepancies. The reasons for discard were primarily that participants selected several age groups per contact, or they systematically filled in the same number of contacts for all entries. Overall, data are presented for 568 (50% of 1138; 54% female) useable diaries from participants with a mean age of 23 years (range 0.1–84.9 years). See Texts S2 and S3 (raw data and data dictionary, respectively).

Table 1 provides data on some baseline characteristics of the 568 diary participants. The majority of the participants lived in Roka location (26%), with Tezo and Ngerenya providing the smallest proportion of participants. More than two-thirds had less than 4 years of education, and 349 (61%) of the total required a shadow. Half of the participants were unemployed, while a quarter were students. The majority (96%) of the participants preferred the picture to the text diaries. During the exit interview, only 8 of the participants reported having not fully understood how to keep the diary, while the most common issue raised by the shadows was the difficulty in following the selected participant wherever they went. Out of 33 participants who reported an induced behaviour change, 27 had a shadow.

**Table 1:** Baseline characteristics of 568 diary-keeping participants from Kilifi Health and Demographic Surveillance System, Kenya.

Variable		Number, n(%)
Location	Kilifi Township	110 (19.4)
	Tezo	87 (15.3)
	Ngerenya	86 (15.1)
	Roka	151 (26.6)
	Matsangoni	134 (23.6)
Number of years of education	≤4	374 (65.8)
	5–8	144 (25.4)
	9+	50 (8.9)
Diary type preference	Pictorial	545 (96.0)
	Text	23 (4.0)
Diary keeper	Participant	220 (38.7)
	Shadow¥	348 (61.3)
Participant's occupation╞	Student	142 (25.0)
	Employed	137 (24.1)
	Unemployed§	286 (50.4)
Difficulty in filling diary╞	Yes	8 (1.4)
	No	554 (97.5)

¥ 2 primary school students out of 222 participants aged <10 years required two shadows; one at home (parent) and at school (teacher).

╞ Missing records as a proportion of all 568 participants: participant occupation (3, 0.5%); difficulty in filling in diary (6, 1.1%).

§ Unemployed: these include children <6 years, unemployed participants (62% female), pre-school children and retired individuals.

The characteristics of the persons contacted by the diary participants are given in Table 2. The largest proportion of contacts was with siblings (40%) and other relatives (34%), with participants recording only 7% of contacts with parents. While 63% of contacts were with family members (parents, spouses, children and siblings), only about a third (28%) shared the same household as the participant (note that a household frequently includes more than one related family living in different dwellings but within the same compound). Additionally, a third of the contacts slept in the same room as the participants, and out of these two-thirds shared a bed with the participant. Of the total number of people contacted, only 5% were unknown. We do not present any data on the tallies of repeat encounters of contacts.

**Table 2:** Baseline characteristics of 10,042 contacts by participants in a diary study in the Kilifi Health and Demographic Surveillance System, Kenya.

		Contacts (%)
**Relationship to participant^╞$^**	Parent	707 (7.0)
	Sibling	3,985 (39.7)
	Child	1,517 (15.1)
	Spouse	118 (1.2)
	Other relative	3,411 (34.0)
	Other	106 (1.1)
**Live in same house**	Yes	2,855 (28.4)
	No	7,187 (71.6)
**Sleep in same room**	Yes	909 (31.8)
	No	1,924 (67.4)
**Sleep in same room**	Yes	597 (65.7)
**and share bed**	No	312 (34.3)
**Ever met the contact before?**	Yes	9,290 (92.5)
	No	454 (4.5)
**Frequency of meeting** □	Daily	7,287 (78.4)
	Regularly	1,486 (16.0)
	Often	343 (3.7)
	Rarely	136 (1.5)

╞ Missing records as a proportion of the total contacts 10,042): Relationship to participant (198, 2.0%); Sleep in same room (22, 0.8%); Ever met the contact before (298, 3.0%); Frequency of meeting (38, 0.4%).

$ While 63% of contacts with family members (parents, spouses, children and siblings),only 28% live in the same household. Members of the same family could be living in different households and share a common compound (homestead).

□ Frequency of meeting: daily (on a day-to-day basis); regularly (more than four times a week); often (once or twice a week); rarely (once or twice a month).

### Contact rates

**Figure 2:**
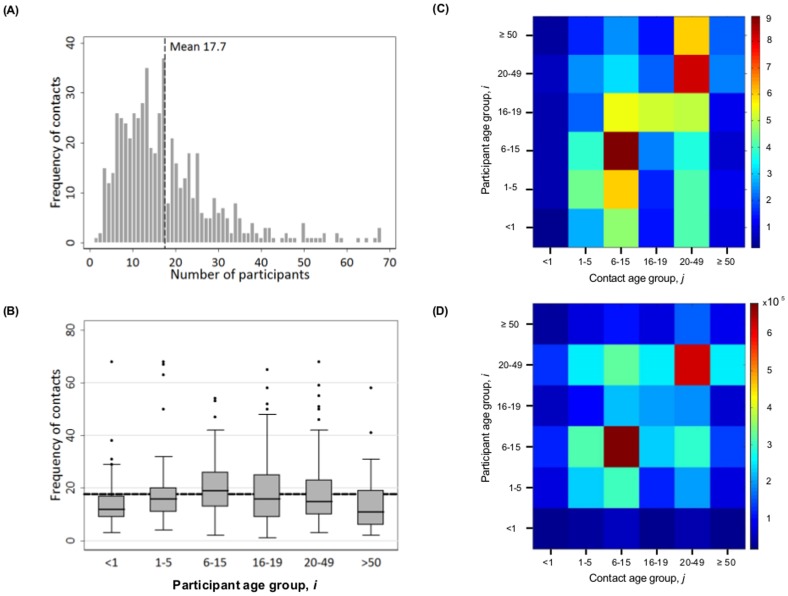
Contact mixing patterns. Part A: Distribution of overall number of contacts (with mean shown as a dashed line). Part B: Mean (dashed line) contact rate per person per day, with boxplots showing median (centre line) and interquartile range (IQR) of contact rates per age group per day. Part C: Contact rate surface (heat map) expressing the mean number of contacts between an individual participant in each age group 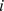 with individuals in each age group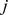. Part D: Population level numbers of contacts per day within and between age groups (estimated from the matrix defined in (C) scaled by the age-specific resident population size).

**Figure 3:**
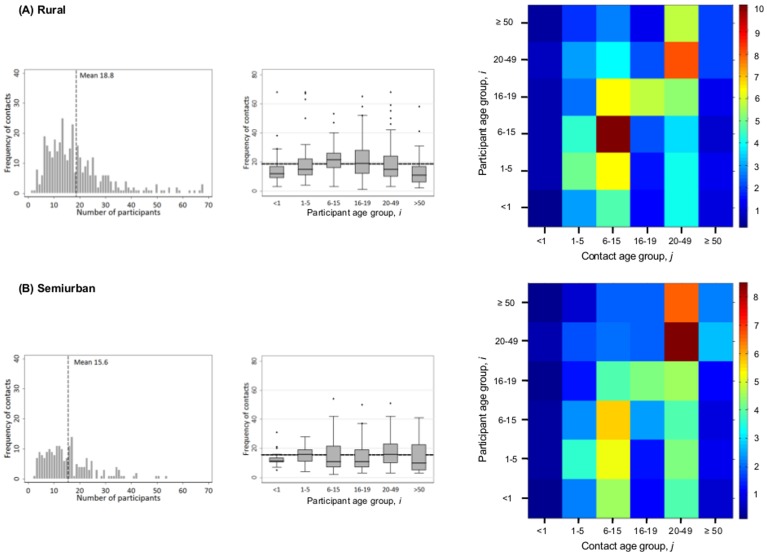
Age specific contact matrices. Mixing patterns for 371 participants in rural areas (Part A) and 197 participants in semiurban areas (Part B). The description of the images, from left to right, follows that in Figure 2 Parts A, B and C, respectively.

A total of 10,042 contacts were recorded in the diaries by the 568 participants. Each participant recorded an average of 17.7 (95% CI 16.7–18.7) contacts per day (Part A of Figure 2). We found that primary school aged children in the KHDSS had the highest contact rate (20.1, 95% CI 18.0–22.2) compared to the rest of the population, with infants and the elderly recording the lowest contact rate at 13.9 (95% CI 12.1–15.6) and 13.9 (95% CI 11.3–16.5) respectively (Part B of Figure 2, Table 3). There was strong evidence that the difference in the age specific mean contact rates was not due to chance (ANOVA F = 4.67, df = 5, p = 0.0003, Table 3). Shadows recorded fewer contacts compared to participants who kept diaries for themselves (16.3 vs 19.9 respectively, ANOVA F = 12.8, df = 1, p = 0.0004). Further analysis by age revealed that this difference was significant in ages 15–19 (p = 0.02) and 20–49 (p = 0.01) years. When stratified by residence, participants in the rural areas reported higher mean number of contacts (18.8/person/day, Part A of Figure 3) compared with their semiurban counterparts (16.5/person/day, Part B of Figure 3. ANOVA F = 9.86, df = 1, p = 0.002, Table 3). In the rural areas, significantly lower contact rates were recorded by shadows compared with participants with self-kept diaries (17.0 vs 22.4, ANOVA F = 15.5, df = 1, p = 0.0001); however, no such difference was observed in the semiurban areas. Similar analysis revealed no evidence that the mean number of contacts recorded differed by sex (p = 0.85), weekend versus weekday (p = 0.72), or season (p = 0.87) (Table 3).

**Table 3:** Mean number of contacts per day stratified by gender, age group (years), presence of shadow, season, residence, days of week of 568 diary participants from the Kilifi Health and Demographic Surveillance System, Kenya.

Category/Covariate		Total participants (n, %)	Mean (95% CI‡) number of people contacted per participant per day (D/n)	P-value
Overall		568	17.7 (16.7–18.7)	
Gender	Male	262	17.6 (16.1–19.1)	
	Female	306 (54%)	17.8 (16.5–19.0)	0.85
Participant	<1	86 (15%)	13.9 (12.0–15.7)	
age group	1–5	93 (16%)	17.6 (15.3–19.9)	
	6–15	98 (17%)	20.1 (18.0–22.2)	
	16–19	91 (16%)	19.4 (16.6–22.1)	
	20–49	139 (25%)	18.9 (16.8–21.1)	
	≥50	61 (11%)	13.9 (11.2–16.6)	0.0003
Shadow	Yes	349	16.3 (15.2–17.4)	
present	No	219 (39%)	19.9 (18.1–21.7)	0.0004
Season$	Dry	212	17.6 (15.9–19.3)	
	Wet	356 (63%)	17.1 (16.6–18.9)	0.87
Location&	Rural	371	18.8 (17.5–20.1)	
	Semiurban	197 (35%)	15.6 (14.2–16.9)	0.002
Day of week	Weekend	168	17.9 (16.2–19.7)	
	Weekday	400 (70%)	17.6 (16.4–18.7)	0.72

‡ 95% CI: 95% confidence intervals derived from 2,000 bootstraps.

$ Season: Dry  =  January, August, December; Wet  =  September – November

& Location. Rural: Ngerenya, Roka, Matsangoni; Semiurban: Kilifi Township, Tezo.

### Age group specific mixing patterns

Figure 2 Part C shows a heat map of mean age specific contact rates between participants in each age class (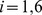; x-axis) stratified by contacted age group (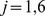; y-axis). The corresponding data table and confidence intervals are presented in Table 4. Furthermore, Table S2 shows the age specific total contacts per day by participants with each contact age group. The effect of weighting for rural–semiurban bias in sampling on the estimated contact rates was found to negligible (not shown) and hence we present the unadjusted estimates (contact matrices in Part A and B, respectively, in Figure 3).

**Table 4:** Age group specific contact rates with 95% CI‡.

		Contact age group*					
		<1	1–5	6–15	16–19	20–49	≥50
**Participant age group** * **(years)**	<1	0.2 (0.1–0.3)	2.7 (2.3–3.2)	4.6 (4.0–5.4)	1.3 (1.1–1.7)	4.0 (3.4–4.7)	1.0 (0.7–1.2)
	1–5	0.5 (0.4–0.7)	4.4 (3.8–5.2)	6.0 (5.1–6.9)	1.5 (1.2–1.8)	4.1 (3.5–4.7)	1.1 (0.9–1.4)
	6–15	0.6 (0.4–0.7)	3.8 (3.2–4.4)	8.9 (7.9–10.1)	2.3 (1.9–2.7)	3.6 (3.1–4.2)	0.9 (0.7–1.1)
	16–19	0.5 (0.3–0.7)	2.0 (1.6–2.5)	5.5 (4.6–6.4)	5.2 (4.4–6.1)	5.0 (4.2–5.8)	1.1 (0.9–1.4)
	20–49	0.7 (0.5–0.8)	2.5 (2.1–2.9)	3.1 (2.7–3.6)	2.1 (1.8–2.5)	8.2 (7.3–9.3)	2.3 (1.9–2.6)
	≥50	0.4 (0.2–0.6)	1.5 (1.1–2.0)	2.5 (1.9–3.1)	1.4 (1.0–1.9)	6.0 (4.8–7.4)	2.1 (1.6–2.7)

‡ Confidence intervals based on 2,000 bootstraps.

^*^Age group in years.

Figure 2 Part C highlights three key features. Overall, there is a strong diagonal element, indicating high contact rates between individuals in the same age groups (assortative mixing) relative to the average. The highest contact rates were within the 6–15 year age group (8.9, 95% CI 8.4–10.5), that is primary-to-primary school children; and adult-to-adult with 8.2 (95% CI 7.2–9.1) contacts per day. The lowest contact rates were infant-to-infant (0.2, 95% CI 0.1–0.3). Second, in general, relatively high contacts rates were recorded by participants of all ages with primary school children and with adults (20–49 years of age). Third, there is an absence of clear symmetry in mixing by reciprocal age groups. For example, the contact rate of 6–15 years old children with 16–19 year olds is estimated to be 2.3 contacts per day, whereas the rate of contact between 16–19 years olds with 6–15 years olds is over twice that at 5.5 contacts per day (Table 4). This is a reflection of the differences between age groups in actual population size. For example, within KHDSS there were 78,805 registered residents aged 5–14 years, compared with 22,440 aged 16–19 years (Table S3). Multiplying each of the rates (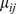) in Figure 2 Part C (Table 4) by the resident population of each participant age group (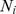) yields the contact matrix shown in Part D of Figure 2 that demonstrates much closer reciprocity of between age group total numbers of contacts. This figure also reveals more clearly relatively high inter-generational contact number, e.g. between school and adult age groups. Comparison of the patterns of contacts between the semiurban and rural population samples is shown in Figure 3. In the rural areas, the highest level of assortativeness is observed among people in the age range 6–14 years. In addition, high levels of mixing are observed between children aged 6–14 years and those aged 1–5 and 15–19 years. By contrast, adults in the semiurban areas have the highest assortative contact rates compared to other age groups with high between group contacts rates mainly occurring between adults and the elderly.

## Discussion

We report estimates of daily physical contact rates within and between different age groups in a rural coastal Kenyan population. On average individuals made 17.7 (95% CI 16.7–18.7) contacts per person per day, with highest rates observed for primary school children aged 6–15 years (20.1, 95% CI 18.0–22.2). Assortative mixing was conspicuous, particularly amongst school-going child age group (6–19 years) and also among the adult age group (20–49 years). In addition, there was strong inter-generational mixing (presumably parents and children, or teachers and pupils), but this was most evident once differences in population size by age were accounted for (Figure 2 Part D). Contact rates were higher in rural compared to semiurban areas, with primary school children recording highest rates in the former and adults (including the elderly) recording highest rates in the latter. There was no evidence of a difference by sex, season and day of the week. These data on contact patterns and rates are important for the evaluation of empirically driven mathematical models that aim to inform prevention strategies and policies against the transmission of diseases that spread via direct contact through the respiratory route (e.g. RSV [1], [3], [20]) or faecal-oral route (rotavirus [21]).

We defined a contact as direct skin-to-skin touch, which has particular relevance to the transmission of RSV [22], reduces under-reporting as it is a less frequent event relative to conversation, and simplifies diary entry. The majority of earlier studies defined contacts as both conversation and skin-to-skin touch, with data being collected via self-kept paper diaries [3], [6], household interviews [8], [23] and web-based interfaces [10], [24]. We report higher (physical only) contact rates than previous studies in urban South Africa [7] and rural Vietnam [8], which estimated both physical and non-physical contacts. Reported physical contact rates in the POLYMOD study [3] conducted in 8 European countries are also lower than those reported here. These differences could be due to the definition of a contact and the social construct (sociodemographic patterns in rural-urban areas, differences in household size, etc). This emphasizes the need for further context-specific studies and more so in developing countries where these conditions are different.

The study was designed intentionally to factor out a range of influences which might have a bearing on contact rates, through stratification by (i) time of the year to remove seasonal (dry and wet) variation from, for example, agricultural practices, (ii) location that captures differences in household occupancy and population density on the rural - semiurban continuum, and (iii) day of the week (weekend versus weekday), to avoid possible bias in behaviour over the period of a week and the context of the contact (e.g. school, household, workplace).

Similar to other studies, we report strongly assortative mixing among school children [3], [5], particularly of primary school age. There is also relatively high contact rates between children of all ages and primary school-age children, and cross-generational, hence increasing the probability of spreading infection throughout the population and within the household setting [25]. This has implications for targeted vaccination as emphasised by a recent modelling exercise which predicted that vaccinating school-going children against influenza, in addition to adults, resulted in a two-fold reduction in infections per dose of vaccine compared to targeting those aged >65 years only [4]. On the other hand, in our study infants (ie aged less than 1 year) reported the lowest contact rates, presumably due to mobility limitations, although infants do spend much time carried by the mother or a sibling. This might increase contacts but potentially may not have been recorded as such in this study. Our findings are important for investigating alternative age-dependent vaccination strategies particularly because previous vaccines used in young infants, who experience the highest burden of disease [26], have experienced several obstacles summarised by Collins *et al* [27].

Higher rates of contacts were observed in rural areas compared to semiurban areas. The pattern of contact rates also differed by location type: there was strong assortative mixing rates in children aged 6–15 years and in adults 20–49 years in rural areas whereas in the semiurban area highest rates of mixing was among adults 20–49 and above. Rural areas in the KHDSS show a marked attenuation of young adults, particularly males, into the surrounding semiurban and urban centres [16] mainly for employment and education. Rural residences are also characterised by larger households and a higher proportion of children compared to semiurban areas. Fewer contacts were recorded in diaries by shadows compared with those self-kept by participants, especially for participants aged ≥15 years and those residing in rural areas. These shadows reported having to forego their daily routines to monitor the participants' contact patterns, but mainly for those participants aged less than 5 years. This suggests that older participants did not need active monitoring as they are able to recall their most recent contacts. It also suggests that in general, shadows did not record all contacts that a participant made. However, for older individuals, this bias was likely reduced through an exit questionnaire shortly following diary completion that aimed to elicit non-recorded contacts.

Unlike previous studies [10], [13], [14], [28], no difference was reported in weekend versus weekday contact rates. This could be an indication of more homogenous mixing patterns throughout the week compared to developed countries. For example, the majority of the KHDSS adult population engages in informal employment and subsistence farming that entails working throughout the week. The social structure of the community also involves most of social activities occurring over the weekend, especially wedding and burial ceremonies where individuals congregate for extended periods of time. Furthermore, no differences were observed by season: a surprising result given the local migration of households to tend crops.

### Limitations

Out of the 1,138 selected participants, 50% participated in the study. This resulted in disproportionate under-sampling of the semiurban setting. Exploration of the effect on contact rates by weighted analysis suggested a negligible impact. No other biases were identified arising from low participation. Replacement of all non-participants was not possible due to time constraints imposed by the monthly sampling strategy.

Data was not collected throughout the holiday period (Christmas and New Year holiday, from 23^rd^ December 2011 to 8^th^ January 2012). In this social context, most families congregate in their ancestral homesteads located in the rural areas over the holidays. Contact rates, therefore, do not reflect possible effects due to holiday periods, also including the effect of vacation time for school children. Over 50% of all diaries were completed with the support of a shadow. The use a third party to record contacts clearly has possible implications to the accuracy of data and comparability to records from other age groups. In general, we attempted to limit under-reporting and behavioural changes through pre-training, alarm reminders and exit interviewing. Nonetheless, a small number of the shadows reported being unable to keep track of the participants (mainly children) during the duration of the study.

### Generalizability

This study was conducted along a semiurban-rural transect and spanned two climatic seasons. Kilifi has one of the highest poverty rates in Kenya, and the main seasonal economic activities are fishing, farming, agriculture and tourism [16]. Even though much of sub-Saharan Africa remains predominantly rural, such results are contextual and can only be generalized with high confidence to similar regions along the Kenyan coast where these activities are prevalent. Future studies should aim at characterizing social contact patterns across different spatial regions in Kenya and elsewhere, particularly in the urban setting which is rapidly growing.

## Conclusions

In summary, we present data on contact patterns and rates in a rural coastal location in Kilifi, Kenya. We discuss the novel methods used to collect the data in sub-Saharan Africa (the use of picture diaries, shadows and reminders), as well as how the challenges encountered were minimised. Similar to earlier studies in other regions, age assortative mixing is reported. This is more pronounced in the younger age groups in rural areas, with semiurban areas indicating highest contact rates among the adults. The age-specific contact rates estimated from this study can be used to parameterize mathematical models useful to predict the impact of different vaccination schedules.

## Supporting Information

Figure S1 **Sample paper diary.** Participants recorded each contact person only once with a unique code, indicated their age from the groups shown, and gave a tally of repeat contacts with each person met.
(TIF)

Figure S2 **Demographic questionnaire.** This was used to collect data on participants' and shadow demographic details.
(TIF)

Figure S3 **Sample Exit questionnaire.** This was used to collect data on frequency of meeting the contact (new or common contacts).
(TIF)

Table S1 **Reasons for refusal by location of the Kilifi HDSS, Kenya.**
(DOCX)

Table S2 **Total number of age group (years)-specific contacts per person per day.**
(DOCX)

Text S1 **Sample weights.**
(DOCX)

Text S2 **Raw data used in analysis.**
(CSV)

Text S3 **Data dictionary.**
(CSV)
