# Supplementary figures and images for: Quantifying Age-Related Rates of Social Contact Using Diaries in a Rural Coastal Population of Kenya
Source: PLoS One. 2014 Aug 15;9(8):e104786. doi: 10.1371/journal.pone.0104786 (PMC4134222; doi:10.1371/journal.pone.0104786)

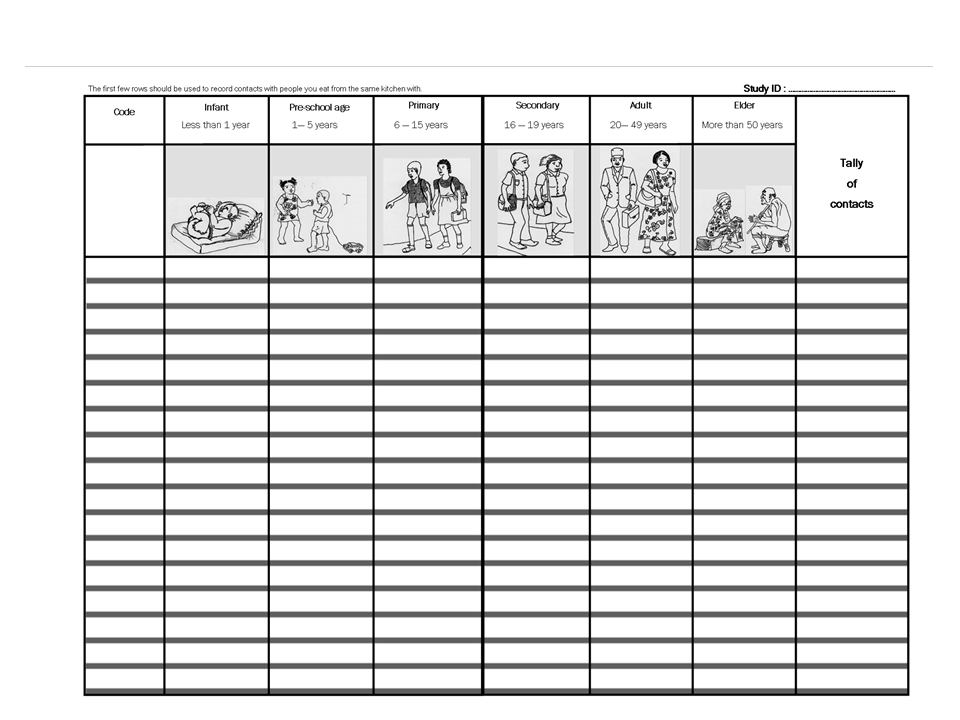

Supplement: Figure S1 — Sample paper diary. Participants recorded each contact person only once with a unique code, indicated their age from the groups shown, and gave a tally of repeat contacts with each person met. (TIF) [file pone.0104786.s001.tif]

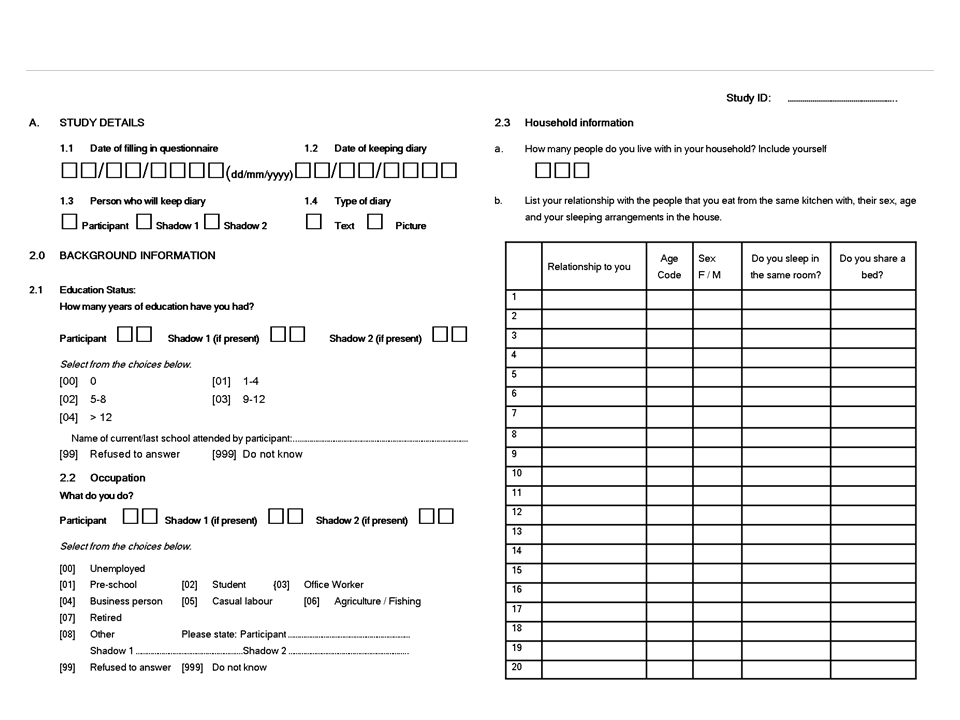

Supplement: Figure S2 — Demographic questionnaire. This was used to collect data on participants' and shadow demographic details. (TIF) [file pone.0104786.s002.tif]

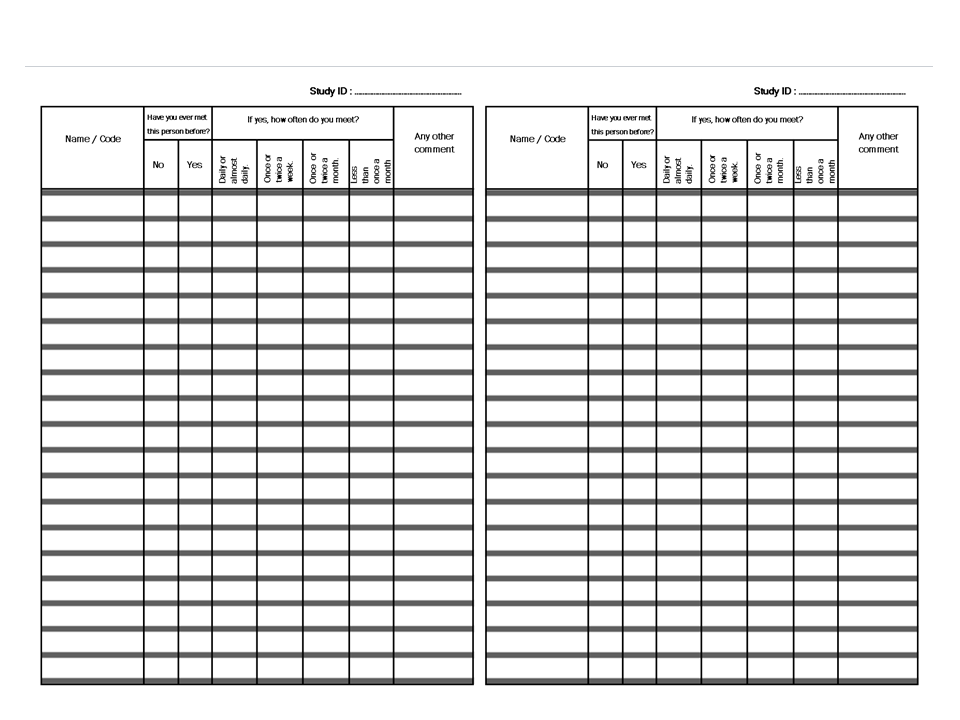

Supplement: Figure S3 — Sample Exit questionnaire. This was used to collect data on frequency of meeting the contact (new or common contacts). (TIF) [file pone.0104786.s003.tif]
