# Supplementary material for: Quantifying Age-Related Rates of Social Contact Using Diaries in a Rural Coastal Population of Kenya
Source: PLoS One. 2014 Aug 15;9(8):e104786. doi: 10.1371/journal.pone.0104786 (PMC4134222; doi:10.1371/journal.pone.0104786)
Supplement: Table S1 — Reasons for refusal by location of the Kilifi HDSS, Kenya. (DOCX) [file pone.0104786.s004.docx]

**Supplementary Information.**

**Table S1.** Reasons for refusal by location by location of the Kilifi HDSS, Kenya.

|  | **Reason for non-consent** | **A** | **B** | **C** | **D** | **E** | **Total** |
| --- | --- | --- | --- | --- | --- | --- | --- |
| Refusal | Hostile | 0 | 0 | 0 | 0 | 1 | 1 |
|  | Silent | 1 | 1 | 0 | 1 | 1 | 4 |
|  | Polite refusal | 28 | 23 | 6 | 9 | 6 | 72 |
| Other | Absent | 14 | 8 | 2 | 6 | 19 | 49 |
| reasons | Migrated | 30 | 5 | 4 | 4 | 14 | 57 |
|  | Temporary away | 27 | 11 | 10 | 17 | 19 | 84 |
|  | Parent failed to give consent | 21 | 3 | 7 | 13 | 2 | 46 |
|  | Untracable | 23 | 3 | 0 | 2 | 1 | 29 |
|  | Withdrawal | 6 | 7 | 0 | 2 | 2 | 17 |
|  | Dropped from study | 9 | 0 | 0 | 0 | 2 | 11 |
|  | Died | 1 | 1 | 1 | 1 | 1 | 5 |
|  | Other | 7 | 2 | 0 | 6 | 6 | 21 |
|  | Unknown | 42 | 15 | 21 | 30 | 11 | 119 |
| Total | | 209 | 79 | 51 | 91 | 85 | 515 |

Note:

Hostile refusal: individual turns violent or shows signs of turning to be hostile/violent.

Polite refusal: individual declines participantion despite understanding requirements of the study.

Silent refusal: individual gives neither hostile nor polite refusal after maximum of three visits

Temporary away: individual is not available at the homestead for more than three consecutive visits

Untraceable: individual is in the HDSS register but cannot be found at location given

Withdrawal: individual opts out of the study

Dropped: when study team deliberately omits individual from study, e.g. unwillingness to cooperate

Died: individual passed away in-between census round and recruitment
