# Supplementary material for: Quantifying Age-Related Rates of Social Contact Using Diaries in a Rural Coastal Population of Kenya
Source: PLoS One. 2014 Aug 15;9(8):e104786. doi: 10.1371/journal.pone.0104786 (PMC4134222; doi:10.1371/journal.pone.0104786)
Supplement: Table S2 — Total number of age group (years)-specific contacts per person per day. (DOCX) [file pone.0104786.s005.docx]

Supplementary Information.

**Table S2.** Total number of age group (years)-specific contacts per person per day

|  |  | **Contact’s age group** | | | | |  |  |
| --- | --- | --- | --- | --- | --- | --- | --- | --- |
|  |  | <1 | 1-5 | 6-15 | 16-19 | 20-49 | >50 | N^8^ |
| **Participant’s age group** | <1 | 18 | 233 | 397 | 116 | 348 | 82 | 10,760 |
|  | 1-5 | 51 | 413 | 555 | 137 | 378 | 103 | 47,433 |
|  | 6-15 | 55 | 371 | 877 | 226 | 355 | 86 | 78,805 |
|  | 16-19 | 45 | 184 | 500 | 474 | 456 | 104 | 22,440 |
|  | 20-49 | 93 | 346 | 436 | 293 | 1,146 | 316 | 94,613 |
|  | >50 | 24 | 92 | 150 | 87 | 366 | 129 | 14,012 |

^8^ N is the number of individuals registered in the KHDSS Enumeration Round 24, collected between 24/05/2011 - 22/09/2011. 8 residents had missing age records.
