# Supplementary material for: Quantifying Age-Related Rates of Social Contact Using Diaries in a Rural Coastal Population of Kenya
Source: PLoS One. 2014 Aug 15;9(8):e104786. doi: 10.1371/journal.pone.0104786 (PMC4134222; doi:10.1371/journal.pone.0104786)
Supplement: Text S1 — Sample weights. (DOCX) [file pone.0104786.s006.docx]

### Supplementary Information.

### Text S1. Sample weights

Sampling weights were used to correct for selection bias between the sample and reference population caused by unequal probability of selection. The distribution of semiurban and rural population within the five locations (reference population) was 60.3% and 39.7% compared to the participant's population distribution at 30.5% and 69.5% respectively. In this study, we were looking for participation that represented the reference population proportionally, that is, 60% versus 40% respectively. Since this proportional distribution was not attained, we weighted the sampled unit by the reciprocal of the probability of selection into the sample. So, if a unit, in our case a participant, is included into the sample with a probability$p_{k}$, then its weight, denoted by$w_{k}$, is given by $w_{k}=\frac{1}{p_{k}}$.

The sums of the weights provide an unbiased estimate of the total number of individuals in the target population. Therefore if the weight associated with individuals$k$ is given as$w_{k}$, then the weighted mean number of contacts per day will be expressed as:$T_{ij}=\frac{\sum_{k=1}^{N_{t}} y_{ij}w_{ij,k}}{\sum_{k=1}^{N_{t}} w_{ij,k}}$. Note that if $w_{ij,k}$ is a constant for all of$k$, then$T_{ij}$ will be equal to$\mu_{ij}$ given earlier.
